# Ultrasound-Assisted Alcoholic Extraction of Lesser Mealworm Larvae Oil: Process Optimization, Physicochemical Characteristics, and Energy Consumption

**DOI:** 10.3390/antiox11101943

**Published:** 2022-09-28

**Authors:** Seyed Mohammad Taghi Gharibzahedi, Zeynep Altintas

**Affiliations:** 1 Institute of Chemistry, Faculty of Natural Sciences and Maths, Technical University of Berlin, Straße des 17, Juni 124, 10623 Berlin, Germany; 2 Institute of Materials Science, Faculty of Engineering, Kiel University, 24143 Kiel, Germany

**Keywords:** insect oil, ultrasound, extraction, diffusion, fatty acid, antioxidant, bioactive compounds

## Abstract

The ultrasound-assisted extraction (UAE) of oil from lesser mealworm (*Alphitobius diaperinus* L.) larvae powders (LMLPs) using ethanol/isopropanol as the superior solvent was optimized. The evaluation of time (9.89–35.11 min), solvent-to-LMLPs (2.39–27.61 *v*/*w*), and temperature (16.36–83.64 °C) showed that the highest extraction efficiency (EE, 88.08%) and in vitro antioxidant activity (IVAA) of reducing power (0.651), and DPPH free-radical scavenging capacity (70.79%) were achieved at 22.5 *v*/*w* solvent-to-LMLPs and 70 °C for 22.64 min. Optimal ultrasound conditions significantly improved the EE than *n*-hexane extraction (60.09%) by reducing the electric energy consumption by ~18.5 times from 0.637 to 0.035 kWh/g. The oil diffusivity in ethanol-isopropanol during the UAE (0.97 × 10^−9^ m^2^/s) was much better than that of *n*-hexane (5.07 × 10^−11^ m^2^/s). The microstructural images confirmed the high efficiency of ethanol-isopropanol in the presence of ultrasounds to remove oil flakes from the internal and external surfaces of LMLPs. The improved IVAA was significantly associated with the total phenolic (4.306 mg GAE/g, r = 0.991) and carotenoid (0.778 mg/g, r = 0.937) contents (*p* < 0.01). Although there was no significant difference in the fatty acid profile between the two extracted oils, ethanol-isopropanol under sonication acceptably improved oxidative stability with lower peroxides, conjugated dienes and trienes, and free fatty acids.

## 1. Introduction

In the last decade, a serious need for new natural sources and extraction processes for edible oils has emerged due to the increased consumer demand. This potential integrated strategy can contribute to overcoming the most important challenges facing the industry for producing future edible oils with excellent quality and health-promoting properties [1,2]. Solvent extraction is usually performed to extract edible oils from a wide span of fruit and vegetable seeds in the industry [3]. *n*-Hexane is the most common consumable solvent to extract lipids due to its easy evaporation, low energy cost, and high selectivity to oils. However, it causes ecological worries and human health problems, such as high flammability and acute toxicity through inhalation, ingestion, and eye or skin contact [4]. Therefore, its substitution with some efficient solvents is considered necessary for the sustainable processing of edible oils. Recently, some emerging technologies have been utilized to extract lipids, such as microwave heating, ultrasonication, high hydrostatic pressure, supercritical/subcritical, and enzyme-assisted extractions. These green technologies usually run at lower extraction times with a minimum solvent usage compared to conventional methods and increase the extraction yield of edible oils with a better oxidative quality [5].

Insect oil is one of the most promising and sustainable sources of edible oil supply. Although the number of edible insects has reached 1900 species, the oil quality of a small number of them, such as yellow mealworm (*Tenebrio molitor*), super worm (*Zophobas morio*), black soldier fly (*Hermetia illucens*), house cricket (*Acheta domesticus*), and Dubia cockroach (*Blaptica dubia*), have been evaluated as substitutions for soybean, fish, and palm oils in aquaculture and poultry [6,7]. Tzompa-Sosa et al. [8] recently examined the sensory attributes of hummus and crackers made of deodorized yellow mealworm oil instead of vegetable oils. The visual appearance of these food products did not change, but lower overall acceptability and hardness compared to vegetable oil-based ones were reported. However, a blend of rapeseed, peanut, and insect oils could improve the flavor features for more consumer preference. Although lesser mealworm (*Alphitobius diaperinus*) is one of the most important species of oil-supplying insects, not enough information on its lipid potential in industrial applications is available. However, the young larvae powders of this insect rich in linoleic, palmitic, and oleic acids have been recently formulated as a new baking constituent to produce high-protein, mineral-dense snacks [9].

Due to the acoustic cavitation, the ultrasonication process can increase the mass transfer and diffusion rate with higher solvent penetration into the solid matrix through the disruption of cell structure [10]. Sound waves during ultrasonic treatment can be propagated into the liquid medium and subsequently forms alternate cycles of compression (high pressure) and rarefaction (low pressure). The generation of cavitation phenomenon and very fine bubbles provides microturbulence and intense collision of particles in the solvent, accelerating the internal and eddy diffusion [11]. This non-thermal process is adaptable in oil industries because of its practical advantages, such as easy operation, fewer costs, higher extraction efficiency (EE), lower oxidation rate, more maintenance of thermo-sensitive bioactive compounds, and fewer negative impacts on the ecosystem [12,13]. 

To propose efficient ultrasound-assisted solvent extraction processes in terms of quantity and quality improvements, it is necessary to develop a collection of mathematical and empirical techniques for optimizing this process even in the presence of complex interactions. Response surface methodology (RSM) is one of the most practical mathematical techniques to optimize industrial processes by relying on appropriate experimental designs [14]. RSM cannot only determine the interactions between processing factors, but also reduces the number of experimental trials, development time, and overall cost [15]. The successful application of RSM to optimize the UAE process to obtain edible oils from plant tissues, such as hemp seeds [16], *Moringa oleifera* leaves [17], *Moringa Peregrina* seeds [18], fresh aerial parts of *Angelica Keiskei* Koidzumi [19], and red mombin seeds (*Spondias purpurea* L.) [20], has been recently performed. In addition, Susanti et al. have recently reported that using the UAE process under the optimal conditions determined by RSM is an efficient method to substitute the conventional technique for the simultaneous extraction of oil and phenolic compounds from red fruit (*Pandanus conoideus*) [21].

To the best of our knowledge, there are no studies found in the literature on the process optimization of ultrasound-assisted extraction (UAE) to obtain lesser mealworm oil (LMO) with the desired physicochemical. As a result, this study aimed (i) to examine the UAE parameters (i.e., solvent-solid ratio, extraction time, and extraction temperature) to achieve the maximum EE and antioxidant capacity of LMO, (ii) to compare the physicochemical properties and fatty acid profile of the oils extracted by *n*-hexane and UAE under the optimal conditions, and (iii) to calculate energy consumption and diffusion coefficients between the two extraction techniques.

## 2. Materials and Methods

### 2.1. Materials, Chemicals, and Reagents

The insect powder of lesser mealworm (*A. diaperinus* L.) containing 59.6% protein, 28.7% fat, 3.7% fiber, 2.7% carbohydrate, and 0.9% salt was purchased from Snack-Insects Co. (Witzeeze, Germany). The total content of protein, fat, and carbohydrate of LMLPs had been determined by the Kjeldahl, Soxhlet, and Bradford (the rapid colorimetrics of phenol-sulfuric acid with the standard of D-glucose) techniques, respectively. Ethanol, isopropanol, *n*-hexane, diethyl ether, methanol, toluene, iso-octane, cyclohexane, anhydrous sodium sulfate, sodium carbonate, iron (III) chloride, potassium hydroxide (KOH), potassium ferricyanide, hydrochloric acid (HCl), trichloroacetic acid (TCA), gallic acid (GA), 1,1- diphenyl-2-picrylhydrazyl (DPPH^⋅^), Folin–Ciocalteu’s reagent, phenolphthalein, phosphate buffer tablets, and vitamin C were purchased from Sigma-Aldrich Chemical Co. (Darmstadt, Germany). All other chemicals used were of analytical grade and obtained from either Sigma-Aldrich or Merck Chemical Co. (Darmstadt, Germany).

### 2.2. Conventional Solvent Extraction

To determine the best organic solvent for the insect oil extraction, lesser mealworm larvae powders (LMLPs) before defatting were dried at 150 °C for 5 min in a vacuum oven (Salvis Lab VC-20 Vacucentre, Rotkreuz, Switzerland) and then sieved through 0.2 mm mesh. The solvent-assisted oil extraction by *n*-hexane, ethanol, isopropanol, and their binary mixtures (1:1 *v*/*v*) was carried out in a solvent:solid ratio of 10:1 *w*/*v* (10.0 g LMLPs), time of 2 h, and a stirring rate of 400 rpm after doing preliminarily experimental investigations. The extraction time differed depending on the boiling point temperature for each organic solvent and azeotrope data for their binary mixtures. The average extraction temperature for *n*-hexane, ethanol, isopropanol, *n*-hexane/ethanol, *n*-hexane/isopropanol, and ethanol/isopropanol were considered to be 65 °C, 75 °C, 80 °C, 57 °C, and 78 °C, respectively. The power consumption for heating and stirring the magnetic stirrer used for the conventional solvent extraction (CSE) was 550 and 8.5 W, respectively. After finishing the extraction process, the liquid–solid mixtures were filtered by passing via a Whatman filter paper using an aspirated Büchner funnel. The residue was re-extracted twice, and the filtrates were combined from the three extraction stages. The solvent was removed by a rotary evaporator (model Hei-VAP Value, Heidolph, Schwabach, Germany) under a vacuum at 40 °C. The collected oil was passed via the layer of anhydrous sodium sulfate placed over a filter paper in a funnel. The oil was lastly weighed and transferred into 10 mL vials, gently flushed with nitrogen gas, capped, and stored at −18 °C until further analysis.

### 2.3. Ultrasound-Assisted Extraction Process

The LMLPs (10.0 g) preheated in a vacuum oven (150 °C, 5 min) with a diameter of 0.2–0.5 mm were mixed with the superior solvent of ethanol-isopropanol (1:1 *v*/*v*) at ratios of 2.39–27.61 *v*/*w* in a 500 mL Erlenmeyer flask. The insect powder–solvent suspensions were treated by ultrasound for 9.89–35.11 min at the frequency of 35 kHz and power equal to 240 W within the ultrasonic bath (type RK 106, Bandelin electronic GmbH & Co. KG, Berlin, Germany) with 75% filled by distilled water at 16.36–83.64 °C. The liquid level in the Erlenmeyer was lower than that of the water bath. The defined levels of time, temperature, and solvent-to-solid ratio were determined based on the experimental design of RSM (Table 1). A digital thermometer was used to check the accuracy of water-bath temperature while the circulation of water controlled the temperature increase in the water bath during the experimental trials. For this, the temperature during extraction was continuously adjusted and maintained at the desired level within ±1 °C by adding hot or cold water at the appropriate level. The crude extracts’ filtration and concentration were also performed per the CSE method.

### 2.4. Extraction Efficiency

Regarding the oil extraction performed from LMLPs with certain fat content, the EE than extraction yield was a better index to show the solvent and UAE capability. The extraction yield can be defined as the ratio of recovered oil weight ($W_{RO}$) to the weight of LMLPs before extraction ($W_{LMLPs},\;10.0\;g$). However, the EE indicates that the percentage of extracted oil concerning the quantity of oil presents in the LMLPs ($W_{OC},\;0.287g\;\mathrm{oil}/g\;\mathrm{LMLPs}$), which can be calculated by the following equation (Equation (1)) [22]:

(1) $$EE\;\left(\%\right)=\frac{W_{RO}}{\left(W_{LMLPs\;}\times W_{OC}\right)}\times 100\;$$

### 2.5. DPPH^⋅^ Scavenging Capacity Assay

The scavenging capacity of DPPH free-radical (*SC_DPPH._*) of extracted LMOs was measured according to the method described by Gharibzahedi et al. [23]. Briefly, 100 mg of each oil sample in 1.0 mL toluene were vortexed with 3.9 mL of the DPPH^⋅^ solution (0.1 mM) in toluene for 30 s, kept in the dark for 1 h at ambient temperature, and finally, their absorbance (*A_s_*) was read at 515 nm using a UV–vis spectrophotometer (Cary 60, Agilent Technologies, Santa Clara, CA, USA). The same procedure was performed in toluene instead of oil sample (*A_0_*) to determine the control absorbance. Vitamin C as a positive control compound was used. The *SC_DPPH_^⋅^* was assessed as follows (Equation (2)):

(2) $$SC_{DPPH}\left(\%\right)=\left(1-\frac{A_{s}}{A_{0}}\right)\times 100\;$$

### 2.6. Reducing Power Assay

The method of Ogbunugafor et al. [24] with small modifications was applied to determine ferric ions’ reducing power (RP). 2.5 mL of each oil sample was initially mixed with 2.5 mL of 1% potassium ferricyanide, incubated at 50 ± 2 °C for 25 min, and then cooled rapidly. An amount of 2.5 mL of 10% TCA solution to the mixture was added, vortexed, and centrifuged at 3000 rpm for 10 min. In the next step, 2.5 mL of the supernatant solution was mixed with 2.5 mL of Millipore water and 0.5 mL of 0.1% iron (III) chloride. Then the absorbance was measured using a UV–vis spectrophotometer at 700 nm.

### 2.7. Single-Factor Exploratory Tests

Single-factor experiments (SFEs) were carried out to assess the effects of the following parameters on EE, SC_DPPH_^⋅^, and RP: UAE time (5–60 min), UAE temperature (10–90 °C), and solvent-to-LMLPs ratio (5:1–45:1 *v*/*w*). According to the single-factor experimental data, the best range of response values in these three experiments were selected to input into RSM’s central composite rotatable design (CCRD).

### 2.8. Experimental Design and Response Surface Optimization

An RSM-CCRD was employed using Design-Expert software (version 8.0., Statease Inc., Minneapolis, MN, USA). Three operating variables involved in the UAE of LMO including time (*X*_1_, 9.89–35.11 min), solvent-to-LMLPs ratio (*X*_2_, 2.39–27.61 *v*/*w*), and temperature (*X*_3_, 16.36–83.64 °C) were optimized to achieve the highest EE, SC_DPPH__⋅_, and RP amounts. Table 1 shows 20 experimental combinations of independent variables at five levels in random order. The above-mentioned range for each studied parameter was determined after performing preliminary trials. Second-order polynomial regression equations were evaluated for the response functions. The generalized response surface model to predict the optimal point is represented as follows (Equation (3)):

(3) $$x={ß}_{0}+\sum_{i=1}^{3}{ß}_{i}X_{i}+\sum_{i=1}^{3}{ß}_{ii}X_{i}^{2}+\sum_{i=1}^{2}\sum_{j=1+1}^{3}{ß}_{ij}X_{i}X_{j}+\varepsilon$$

where *Y* is the dependent variables, ß_0_ is the model constant, ß*_i_*, ß*_ii_*, and ß*_ij_* are the model coefficients, and *ε* is the error. They represent the linear, quadratic, and interaction effects of the variables.

The significance of regression equations was statistically checked. As the significant terms in the model were found by analysis of variance (ANOVA) for each response, insignificant terms were removed in the final model. The quality of all fitted models was assessed based on several statistical parameters such as the coefficient of determination (R^2^), adjusted R^2^ (R^2^_adj_), coefficient of variation (CV), and adequate precision (AP) [25]. Five extra tests were performed to verify the accuracy of the optimal points predicted by the RSM package’s response optimizer. The validity of the fitted models was also confirmed by comparing the actual and predicted data according to Student’s *t*-test using SPSS V.21 (SPSS Inc., Inc., Chicago, IL, USA) software at a significant level of 5%.

### 2.9. Evaluation of Total Phenolic Content

The total phenolic content (TPC) of oil samples was determined based on the modified method of Singleton and Rossi [26]. A calibration curve with the standard of GA (10–100 µg/mL) in methanol was constructed for this test. 400 µL of GA in each concentration with 2 mL of dilution Folin–Ciocalteu’s reagent (1:10) was mixed, and then 1.6 mL of 7.5% sodium carbonate was added. The same experiment with oil samples instead of GA was done to measure the TPC. After vortexing, the mixtures were incubated for 60 min in the dark at room temperature, and their absorbance using a UV–visible spectrophotometer was read at 765 nm. The mean TPC results were expressed as milligrams of GA equivalents per gram of oil (GAE/g oil).

### 2.10. Determination of Total Carotenoid Content

The method described by Naebi et al. [27] was used to assess the total carotenoid content (TCC). For this experiment, 7.5 g of each oil sample in a 25 mL volumetric flask was brought up to the volume with cyclohexane, and then their absorbance was measured by a UV–visible spectrophotometer at 470 nm. The *TCC* (mg/kg) was calculated based on the following formula (Equation (4)):

(4) $$TCC\;\left(\mathrm{mg}/\mathrm{kg}\right)=\frac{\left(A_{470}\times 10^{6}\right)}{2000\times 100\times L}$$

where $A_{470}$ is the absorbance amount at λ = 470 nm, and *L* is the thickness of the spectrophotometer cell (1 cm).

### 2.11. Analysis of Fatty Acids Profile

The AOAC procedure was used to prepare the fatty acid methyl ester (FAME) [28]. At first, LMO (50 mg) dissolved in 4.0 mL of methanolic HCl (0.5 M) was highly vortexed and incubated for 4 h at 50 ± 2 °C. Then, the mixture was immediately cooled down to room temperature and the FAME was purified with 10 mL of *n*-hexane. The layer of anhydrous sodium sulfate was used to dry the clear upper layer containing FAME. Identification and quantification of the extracted FAME profile were carried out using gas chromatography (GC, Agilent 6890, Agilent Technologies, Wilmington, DE, USA) equipped with a Chrome-pack BPX5 capillary column (30 m × 0.25 mm × 0.25 μm) and an ionization mass detector (Agilent 5973N, USA). The flow rate of carrier gas (helium) and the split ratio were 1.0 mL/min and 100.0, respectively. The oven temperature program included: initially set at 60 °C (isothermal for 10 min) and gradually increased from 70 to 140 °C with a 10 °C/min rate. After holding for 10 min at 140 °C, it was increased to 250 °C with a rate of 7 °C/min, and lastly, isothermally kept at 280 °C for 10 min. In addition, the mass detector was set under the following conditions: capillary direct interface temperature of 240 °C, ionization energy of 70 eV, scanning interval of 0.5 s, and mass range of 40–1000 *m*/*z* [29]. The comparison of retention indices of fatty acids with their authentic samples and the mass spectral data available in the library (Wiley-VCH 2001 data software, Weinheim, Germany) contributed to identifying and determining each fatty acid.

### 2.12. Assessment of Physicochemical Properties

The apparent viscosity, specific gravity, and refractive index were assessed by Brookfield rotational viscometer (DV-II+PRO model, Brookfield Engineering Labs., Inc., Middleboro, Brookfield, MA, USA), pycnometer, and Abbe refractometer (Carl Zeiss, model G, Jena, Switzerland), respectively. The browning index (BI) was measured by determining the spectrophotometric absorbance of the diluted mixture of oil with *n*-hexane (1:20, *w*/*v*) at 420 nm [30]. The photometric color index (PCI) of the oil in the visible spectrum (λ = 460, 550, 620, and 670 nm) was estimated by the following formula (Equation (5)) [31]:

PCI = 1.29 (A_460_) + 69.7 (A_550_) + 41.2 (A_620_) − 56.4 (A_670_) (5)

The acid (AV, mg KOH/g), saponification (SV, mg KOH/g), iodine value (IV), peroxide (PV, meq O_2_/kg), and p-anisidine (p-AnV) values of the extracted oils were respectively determined based on the AOCS standard methods of Cd 3a-63, Cd 3-25, Cd 1d-92, Cd 8-53, and Cd 18-90 [32]. The totox value (TxV) is calculated by the formula of 2PV + p-AnV to indicate an oil’s overall oxidation state. The conjugated diene (K_232_) and triene (K_270_) levels based on the standard procedure of ISO 3656:2011 were measured by determining the absorbance of 1% (*w*/*v*) oil solution in cyclohexane at 232 and 270 nm, respectively [33]. The oxidative stability of LMOs was evaluated using the Rancimat (Rancimat 679, Metrohm, Herisau, Switzerland) at an airflow rate of 20 L/h and a temperature of the heating block of 120 °C [34]. The procedure of Wang et al. [35] with small modifications was applied to evaluate the content of free fatty acid (FFA). After dissolving LMO (2.0 g) in ethanol-diethyl ether (50 mL, 1:2 *v*/*v*), the mixture was stirred for 30 min at room temperature and titrated against 0.05 M KOH using the phenolphthalein indicator. The *FFA* content was then calculated using the following formula (Equation (6)):

(6) $$FFA\left(\mathrm{mg}/\mathrm{kg}\right)=\frac{\left(V\times C\times 56.11\right)}{m}$$

where *V* is the volume of KOH exhausted by LMOs in mL, *C* is the concentration of KOH (0.05 M), and *m* is the mass of the oil sample in g (2.0).

### 2.13. Estimation of Diffusion Coefficients

Fick’s second law (FSL) can be used to describe the oil extraction in CSE and UAE processes (Equation (7)) [4]:

(7) $$\frac{\partial C}{\partial t}=D\frac{\partial^{2}C}{\partial X^{2}}$$

where *C*, *t*, *X*, and *D* are the solute concentration, the time (s), the particle thickness (m), and the diffusion coefficient (m^2^/s), respectively.

There were three assumptions, including (i) taking into account insect powders as small spheres, (ii) neglecting the resistance to the external mass transfer due to the vigorous stirring, and (iii) the free-solute solvent at the beginning of the process. The FSL solution for a stirred solution in a limited volume can be done by Equation (8) [36]:

(8) MtM∞=1−∑n=1∞6αα+1exp−Dqn2tα29+9α+qn2+α2

where $M_{t}$ and $M_{\infty }$ are the total oil amount in LMLPs at the time *t* and after an infinite time of diffusion, respectively. α is the mass ratio of the solvent and LMLPs. Furthermore, $q_{n}\;$ is non-zero positive roots of Equation (9):

(9) $$\tan q_{n}=\frac{3q_{n}}{3+\alpha q_{n}^{2}}$$

### 2.14. Calculation of Electric Energy Consumption

The electric energy consumption (EEC) of UAE and CSE processes per gram LMO can be calculated as follows (Equation (10)) [37]:

(10) $$EEC\left(kW.h/g\right)=\left(\frac{P\times t}{m}\right)$$

where *P*, *t*, and *m* are the power consumption (kW), the extraction time (h), and the mass of obtained oil (g), respectively.

### 2.15. Scanning Electron Microscopy

The LMLPs before and after defatting by the CSE and UAE methods were vacuum-dried at 75 °C for 24 h, coated with a thin layer of gold employing a desktop sputtering system, and visualized using the field emission-scanning electron microscopy (FE-SEM, Zeiss Gemini DSM 982, Carl Zeiss Ltd, Oberkochen, Germany) at a 30 μm scale bar under 1000× magnification and an accelerating voltage of 6 kV.

### 2.16. Statistical Analysis

The data were a mean of three experimental replications and subjected to ANOVA using SPSS V.21 software. Significant differences were assessed by Duncan’s test (*p* < 0.05). Pearson’s coefficient performed the correlation analysis between antioxidant activity (RP/SC_DPPH_) and bioactive compounds (TPC/TCC).

## 3. Results and Discussion

### 3.1. Selection of the Organic Solvent

**Figure 1:**
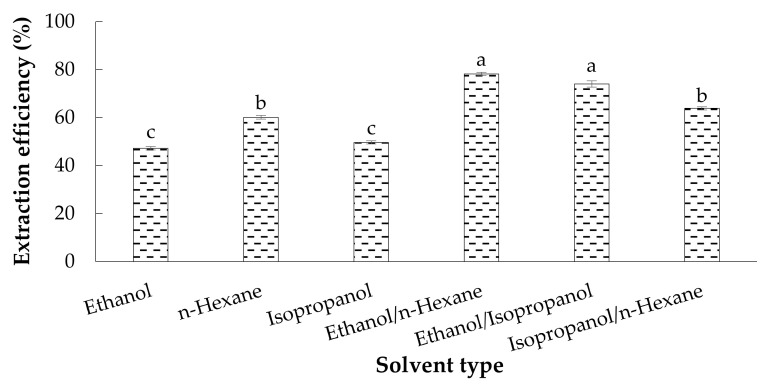
The EE comparison of oils extracted from LMLPs by pure and binary mixed organic solvents. Means with different superscript letters (a–c) in columns indicate the significant statistical difference (*p* < 0.05).

Figure 1 compares the EE of LMOs extracted by the pure organic solvents and their binary mixtures. The results showed the use of two binary mixtures of *n*-hexane/ethanol (78.18%) and ethanol/isopropanol (74.03%) than the other solvents (47.21–63.85%) significantly had more capability to extract oil from LMLPs (*p* < 0.05). As there was no significant difference in the EE between ethanol/isopropanol and *n*-hexane/ethanol, the alcoholic mixture was chosen for the UAE due to the removal of *n*-hexane. In general, the solvent polarity can be determined based on its dielectric constant. Accordingly, polar solvents like ethanol (24.55 C^2^/N·M^2^, 25 °C) and isopropanol (19.92 C^2^/N·M^2^, 25 °C) compared to the nonpolar solvent such as *n*-hexane (1.88 C^2^/(N·M) are more able to extract polar constituents from natural substances, resulting in a higher EE [38,39]. In other words, the difference in oil extraction yields obtained from pure organic solvents (i.e., *n*-hexane, ethanol, and isopropanol) and their binary mixtures can be related to the co-extraction of any polar compounds, lipidic or not, enhancing the mass of extracted oil. Espinosa-Pardo et al. realized that ethanol compared to hexane, had a better ability to increase the extraction yield of corn germ oil due to the extraction of other polar compounds from the lipid matrix [40]. Accordingly, higher EE of *n*-hexane/ethanol may be contributed to the separation of phospholipids by the polar phase (ethanol) and more nonpolar lipids like triglycerides by the non-polar phase (*n*-hexane) [38,41]. Recently, a combination of these two solvents (hexane/ethanol; 1:4 *v*/*v*) has resulted in the highest efficiency in extracting oil from shrimp by-products [41]. The high ability of polar solvents to increase EE of LMOs explains that the alcoholic mixture contained more polar lipids such as mono-glycerides (MAG), di-glycerides (DAG), phospholipids, and lipoproteins) than *n*-hexane or its combination with each of the alcohols.

### 3.2. Single-Factor Experiments

**Figure 2:**
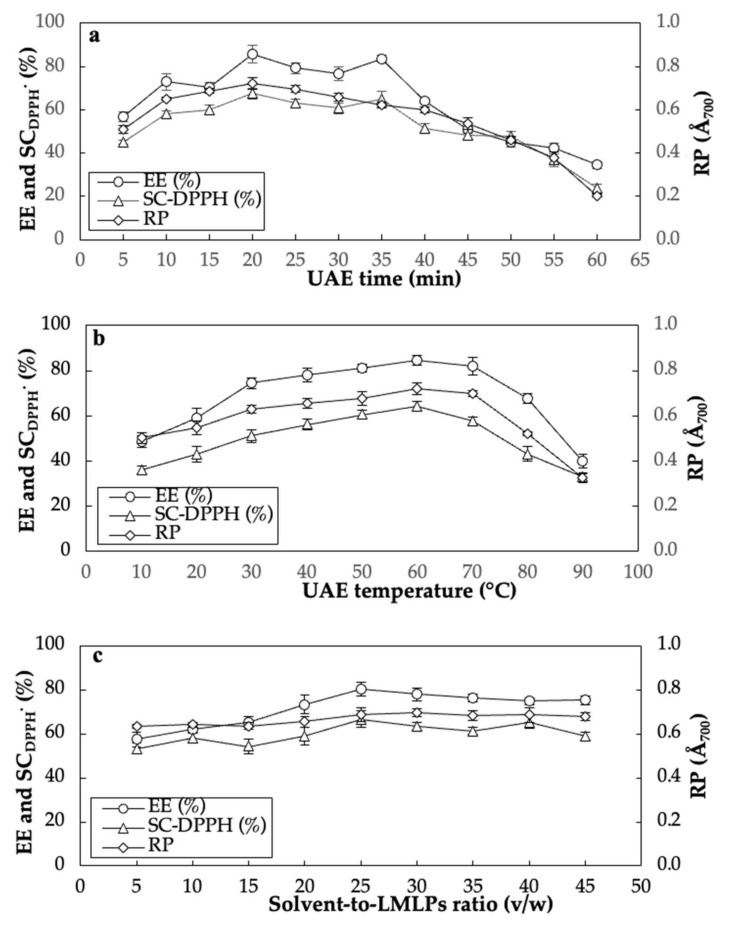
The effect of UAE time (**a**), extracted at 240 W, 65 °C, and 20:1 (*v*/*w*) solvent-to-LMLPs), UAE temperature (**b**), extracted at 240 W, 20 min, and 20:1 (*v*/*w*) solvent-to-LMLPs), and solvent-to-LMLPs (**c**), extracted at 240 W, 20 min, and 60 °C using ethanol/isopropanol on the EE, SC_DPPH_^⋅^, and RP.

The results of SFEs showed that the best ranges of UAE time, UAE temperature, and solvent-to-LMLPs ratio to achieve the maximum EE, SC_DPPH_^⋅^, and RP amounts were 10–35 min, 30–70 °C, and 5:1–30:1 *v*/*w*, respectively (Figure 2a–c). As shown in Figure 2a, the EE rate fluctuated between 72.87 and 85.67% for the insect oil in the time range of 10–35 min. The maximum SC_DPPH_^⋅^ (58.06–64.87%) and RP (0.623–0.722) amounts were also found in the time duration of 10–35 min. However, an increase in the UAE time from 35 to 60 min led to a significant reduction in the EE and antioxidant activity (Figure 2a). An increase in the UAE time increases the effective disruption of the cell walls and leads to a better mass transfer of intracellular products into the solvent. However, a long UAE time can reduce the permeability of solvent into the cell walls because of over-suspended impurities. 

It was found that the longer exposure of lipid substances in the extract can degrade them during the oxidation processes and decrease their antioxidant ability [42]. Figure 2b illustrates the effect of UAE temperature on the EE and antioxidant activity of *A. diaperinus* oil. A significant increase was observed in the EE (74.48–84.56%), SC_DPPH_^⋅^ (51.11–64.12%), and RP (0.628–0.721) amounts when the UAE temperature increased from 30 to 70 °C. However, these quantitative and qualitative properties meaningfully dropped at temperatures above 70 °C and less than 30 °C (Figure 2b). Increased UAE temperature probably improves the solubility and viscosity of *A. diaperinus* larvae lipids and bioactive compounds in ethanol/isopropanol.

Nevertheless, the yield reduction of EE at higher temperatures may be attributed to the intensity reduction of the cavitation phenomenon. Under this condition, the cohesive force developed by the count of cavitation bubbles can decrease the tensile strength of the liquid. Accelerated evaporation at high temperatures also can facilitate the formation of free radicals to oxidize available lipids in the extract [43,44]. The solvent-to-LMLPs ratio effect on the EE and antioxidant activity of the extracted insect oil is exhibited in Figure 2c. An increase in this ratio from 5 to 30 *v*/*w* increased the EE and antioxidant activity. This fact can be owing to the mass transfer improvement of oil and bio-functional constituents at a larger concentration difference between the liquid (ethanol/isopropanol) to solids. No significant change in the EE and antioxidative responses in the solvent volume of more than 30 *v*/*w* was detected as the mass transfer of oil globules and bioactive compounds (e.g., carotenoids and phenolics) are more confined to the solid interior. A similar result was also reported by Zhang et al. [45] and Zhang et al. [46] for the UAE of edible oils from flaxseeds and autoclaved almond powders, respectively.

### 3.3. Fitting the Mathematical Models

Highly significant second-order polynomial models (*p* < 0.0001) with an insignificant lack-of-fit were satisfactorily fitted to predict the independent variables based on the multiple linear regression analysis of the experimental data (Table 2). The obtained models for the EE (Y_1_), SC_DPPH.__⋅_ (Y_2_), and RP (Y_3_) in terms of the experimental (uncoded, Table 1) data are given as follows (Equations (11)–(13)):

Y_1_ = 73.37 + 6.28X_1_ + 11.40X_2_ + 10.37X_3_ − 1.97X_1_X_2_ − 2.31X_2_X_3_ − 4.25X_3_^2^. (11)

Y_2_ = 61.72 + 3.31X_1_ + 5.74X_2_ + 1.93X_3_ − 3.39X_1_X_2_ − 2.09X_1_X_3_ − 3.02X_2_X_3_ − 4.63X_1_^2^ + 1.84X_2_^2^ + 2.63X_3_^2^ (12)

Y_3_ = 0.51 + 0.039X_1_ + 0.094X_2_ − 0.036X_2_X_3_ − 0.064X_1_^2^ + 0.044X_3_^2^. (13)

The quadratic models (*p* < 0.0001) for EE, SC_DPPH._, and RP responses were fitted with high R^2^ (0.9348–0.9870), R^2^_adj_ (0.8760–0.9752), and AP (15.41–33.86) accompanied by low CV (3.31–8.35) values, signifying the adequacy of fitted models and experiments’ high precision and reliability. Overall, a high R^2^_adj_ proves that non-significant terms have not been included in the model. In addition, the AP measures the signal-to-noise ratio, while a ratio greater than 4.0 would be desirable [47]. Table 1 shows that the polynomial regression models sufficiently cover the experimental range of each response variable.

### 3.4. Effects of Independent Variables on the EE

**Figure 3:**
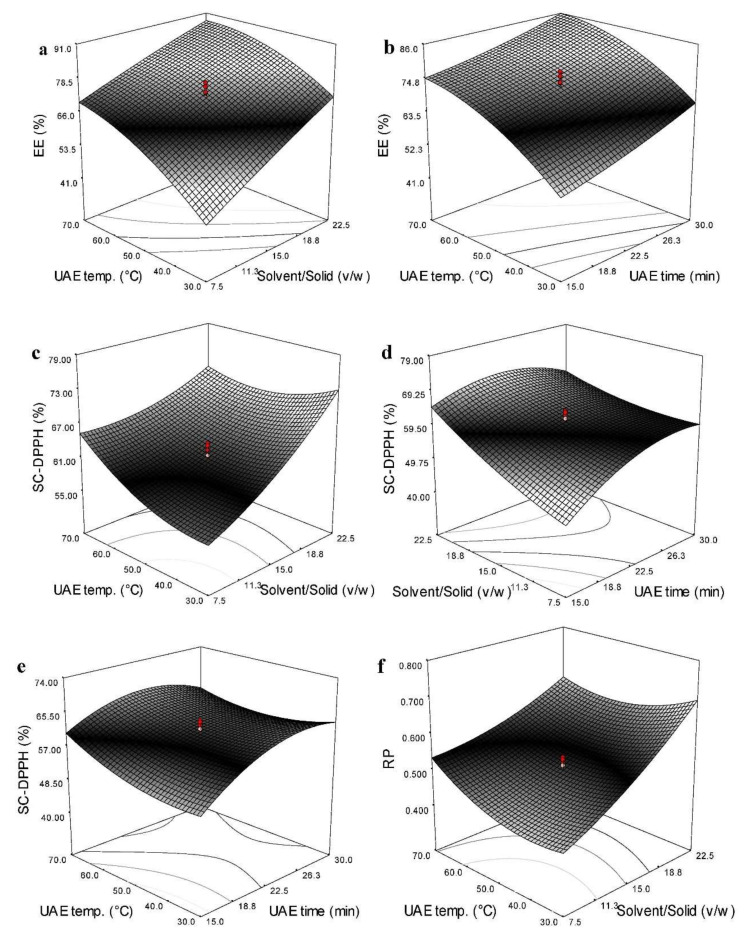
Response surface plots of significant mutual interactions on the EE (**a**,**b**), SC_DPPH_ (**c**–**e**), and RP (**f**) of LMO extracted by UAE using ethanol/isopropanol.

Table 2 illustrates that, among the three independent variables, solvent-to-LMLPs ratio exerted the maximum significance on the EE (*p* < 0.0001; SS = 1773.81) followed by UAE temperature (*p* < 0.0001; SS = 1469.62) and UAE time (*p* < 0.0001, SS = 539.28). The only quadratic effect of UAE temperature was highly significant among the quadratic terms. The mutual interaction between solvent/solid ratio and UAE time and solvent/solid ratio and UAE temperature was found to be significant (*p* < 0.05, Table 2). Figure 3a,b show that an increase in all the independent variables in the studied range resulted in increased EE. The individual optimum condition showed that maximum EE (92.42%) was predicted to be obtained in the UAE under 29.88 min time, 22.50 (*v*/*w*) solvent/solid ratio, and 64.42 °C temperature.

The cavitation phenomenon can be increased with an increase in the applied sonication time. More ultrasonic waves at prolonged times generated high-shear gradients by causing microstreaming to form collapsing bubbles in the vicinity of the cell membrane. As a result, more disruption of cellular membranes increased the contact surface between oil flakes and alcoholic solvents to accelerate the solvent penetration into insect substances for the oil liberation from cells into the solvent with an improvement in the mass transfer rate [48]. The increased mass transfer at higher temperatures may be attributed to the reduction of viscosity and density of solvent mixtures. In addition, it was possible to develop a cohesive force to decrease the tensile strength of the liquid by increasing the count of microbubbles under cavitation with low vapor pressure, reducing the solvent viscosity [44]. The reduced EE at extremely high UAE temperatures may be related to the isomerization and degradation of polyunsaturated fatty acids and other oily constituents [49]. Increasing the EE with an increase in the solvent/solid ratio was based on the principle of mass transfer because the concentration gradient between solids and liquids is considered a driving force for mass transfer [50,51].

### 3.5. Effects of Independent Variables on the Antioxidant Activity

Table 2 shows that the linear, quadratic, and interaction effects of all the independent variables on the SC_DPPH_^⋅^ were significant. However, the RP was significantly affected by the linear effects of UAE time (*p* < 0.01) and solid/solvent ratio (*p* < 0.0001). In addition, the quadratic effects of UAE time and temperature were significant on the RP. Only the significant cross term on the RP was found between the solid/solvent ratio and UAE temperature (Table 2). Interestingly, the most significant effect on the SC_DPPH_ and RP belonged to the main effect of the solid/solvent ratio. Figure 3c–f illustrate that the SC_DPPH_ and RP were significantly boosted by increasing the UAE temperature and solvent-to-LMLPs ratio. However, RP was reduced at higher extraction times. From the individual optimization data, a combination of 24.87 min time, 22.50 (*v*/*w*) solvent/solid ratio, and 31.5 °C temperature was predicted to achieve the highest antioxidant activity (SC_DPPH_^⋅^ (73.24%) and RP (0.705)) of LMO extracted by the UAE.

Free radicals can be formed when hydrogen atoms are lost from double bonds in the molecular structure of unsaturated fatty acids. The inhibition of free radicals with some natural antioxidants such as phenolics, carotenoid pigments, and tocopherols can spontaneously retard or prevent the initiation of the chain reaction and, subsequently, lipid oxidation. Therefore, more protection of these bioactive compounds during the UAE process can improve the antioxidant activity of LMO because the donation of hydrogen atoms or the transfer of electrons to free radicals can contribute to the production of stable radicals by interfering with the propagation reaction [52]. Samaram et al. [53] also found that oil-soluble antioxidants from papaya seeds were more recovered in warm media and additional movements. Increasing the antioxidant activity at higher temperatures may be attributed to the higher solubility of bioactive compounds such as carotenoids and phenolics [54]. An increase in the content of phenols of grape seeds with increasing the UAE temperature from 33 to 67 °C was earlier reported [55]. High solvent ratios probably intensify the capacity of organic solvents to transfer antioxidant ingredients with an increment in the diffusion rate by reducing the viscosity [52,53]. Reducing the antioxidant capacity at longer extraction times can be due to the degradation of unstable minor compounds such as phenols due to the collapse of cavitation bubbles and the generation of short-lived localized hot spots with extremely high local temperature and pressure [56].

### 3.6. Optimal Conditions and Verification of the Models

The overall optimum region with the highest EE (88.08%), SC_DPPH_ (70.79%), and RP (0.651) of LMO was obtained in the UAE under 22.64 min time, 70 °C temperature, and the solvent-to-LMLPs ratio of 22.5 *v*/*w* using the RSM package’s response optimizer. Five runs of additional confirmation tests under the optimal conditions showed that the corresponding experimental values for EE, SC_DPPH_^⋅^, and RP were 89.4 ± 1.8%, 71.3 ± 0.8%, and 0.663 ± 0.016, respectively. There was no significant difference found between the experimental and predicted values. Therefore, the second-order polynomial models presented in this study were efficient in optimizing the operating parameters involved in the UAE of LMOs using the alcoholic mixture of ethanol/isopropanol.

### 3.7. Comparison of Ultrasound and n-Hexane Extraction Methods

#### 3.7.1. Process Efficiency

**Figure 4:**
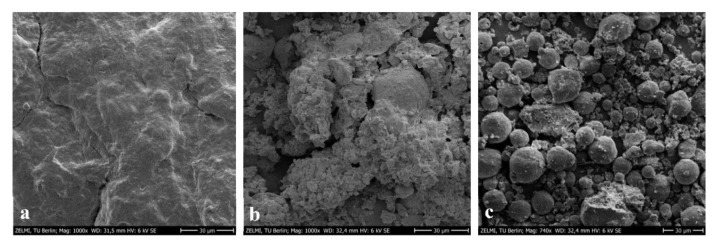
The SEM images of untreated LMLPs (**a**) and substances defatted by CSE (**b**) and UAE under optimal conditions (**c**).

Results showed the EE of LMO obtained under the optimal UAE using ethanol/isopropanol (89.41%) was much better than that of CSE with *n*-hexane (60.09%) (*p* < 0.05). The calculated diffusion coefficients confirmed that LMO during ultrasonication was extracted with a more velocity (0.97 × 10^−9^ m^2^/s) compared to the CSE (5.07 × 10^−11^ m^2^/s) (Table 3). IN addition, the SEM images visualized in Figure 4 show that the UAE and CSE drastically changed the microstructure of untreated LMLPs with a smooth and intact surface. The presence of solvents during stirring and ultrasonication led to the breakdown of cell walls and surface perforations with different intensities and extensions. In the CSE, the sample microstructure was partially damaged, whilst the microstructure by acoustic waves due to the cavitational effect was destroyed, with many irregular pores (Figure 4). Thus, the mixture of ethanol/isopropanol could easier penetrate the sample structure to extract lipids with a higher mass transfer rate. 

A comparison in the EEC reveals that the diffusion, disruption, and leaching out of lipids in UAE (0.035 kW.h/g) needed much lower energy to be implemented than the CSE (0.647 kW.h/g) (*p* < 0.01, Table 3). Accordingly, the EEC of CSE was 18.48 times more than that of the UAE. This result was consistent with the findings of Ideris et al. [57], who suggested the UAE method saves both time and energy for oil extraction from *Canarium odontophyllum* kernels. The EEC reduction is related to the decrease in consumed power. The applied power of the UAE (240 W) was much less than that of the conventional method (558.5 W), based on Equation (10), the EEC reduction can be justified by decreasing the extraction time in the UAE process. Ultrasonic waves produce vibrations for the development of voids to rapidly transfer energy to solid particles immersed in the extraction. Moreover, cavitation bubbles at a short time grow closer to the solid surface and collapse at a higher amplitude forcing the cell wall to rupture, further accelerating the transfer of desired compounds trapped inside into the solvent medium [10,58,59].

#### 3.7.2. Bioactive Compounds and Antioxidant Activity

The SC_DPPH_ and RP amounts of lipids extracted by the CSE were 60.10% and 0.517, respectively, which were significantly lower than those of lipids extracted by the UAE (Table 3). The TPC and TCC for lipids extracted by CSE were 3.652 mg GAE/g and 0.645 mg/g, respectively, whereas the corresponding values for UAE were 4.306 mg GAE/g and 0.778 mg/g, respectively (*p* < 0.05). The SC_DPPH_^⋅^ of lipids extracted by CSE and UAE showed a significantly positive association with TPC (r = 0.968–0.991) and TCC (r = 0.908–0.937). Furthermore, a strong correlation was identified between the RP and TPC in both CSE (r = 0.856, *p* < 0.01) and UAE (r = 0.921, *p* < 0.01). However, no significant correlation was found between RP and TCC of extracted lipids.

Although there is no report on the type of carotenoid compounds present in oils extracted from lesser mealworm larvae, the presence of retinol, lutein, zeaxanthin, β-cryptoxanthin, α-carotene, *cis*-β-carotene, and *trans*-β-carotene in locust (*Locusta migratoria*), melon bug (*Aspongopus viduatus*), and black soldier fly (*H. illucens*) larvae oils were evidenced [60]. Moreover, Nino et al. showed that the main compounds identified in the phenolic profile of edible house cricket (*A. domesticus*) extracts were 4-hydroxybenzoic, *p*-coumaric, ferulic, and syringic acids. Other phenolic compounds with strong free-radical scavenging ability were quinic, gallic, chlorogenic, caffeic, sinapic, and 2-hydroxybenzoic acids [61]. More presence of carotenoids and phenolics in lipids extracted by the optimal UAE process can be a result of the stronger deterioration of plant tissues through the formation of cavitational bubbles in liquids with a lower surface [55,56,57]. Shorter times in the UAE also can maintain bioactive compounds against chemical alterations like hydrolysis, isomerization, and oxidation [12].

#### 3.7.3. Fatty Acid Composition and Physicochemical Properties

The GC-MS analysis showed that the linoleic (C18:2n-6, 30.18–31.52%), oleic (C18:1Δ9c, 28.01–29.54%), palmitic (C16:0, 26.32–28.65%), stearic (C18:0, 7.56–7.68%), and α-linolenic (C18:3n-3, 2.01–2.05%) acids were the most dominant fatty acids in the LMO composition. Myristic (C14:0), gondoic (C20:1Δ11), arachidic (C20:0), palmitoleic (C16:1), lauric (C12:0), and caproic (C6:0) acids in very small levels were also found. There was no significant difference in the fatty acid profile of lipids extracted by CSE and UAE methods (Table 3). Oleic, linoleic, and palmitic acids were also the major fatty acids in lipids extracted from yellow mealworm, buffalo mealworm, house cricket, and Dubia cockroach [62]. Similar findings were reported by Roncolini et al. [9], who found that the main fatty acids of *A. domesticus* were C18:2 (33.66%), C18:1 (28.97%), C16:0 (24.98%), and C18:0 (7.23%). Jalili et al. [63] also showed that the fatty acid profile was hardly affected by UAE as there was no significant difference in the fatty acid composition of canola seed oils extracted by soxhlet and UAE. Moreover, significant differences in none of the physical properties of LMO, such as apparent viscosity, specific gravity, refractive index, BI, and PCI between the two oils, were detected. Except for the SV, lipids extracted by the UAE showed lower AV, PV, p-AnV, TxV, K_232_, K_270_, and FFA levels compared to CSE (*p* < 0.05, Table 3). Hence, more thermal stability was determined for LMOs extracted by UAE under optimal conditions than CSE. 

The IT evaluated using the Rancimat for LMOs extracted by CSE and UAE was 17.22 and 19.56 h, respectively (*p* < 0.05). The AV of oil indicates its deterioration degree because it measures the number of free carboxylic acid groups [64]. Lower AV of LMO extracted by UAE shows that this oil had better quality due to a shorter time and a higher solvent/solid ratio [65]. Longer times in the CSE process probably escalated the decomposition and oxidation of triacylglycerols, resulting in a rise in the FFA content and other oxidative parameters [64,65]. Better oxidative properties along with the high similarity of fatty acids profile showed that the UAE with ethanol/isopropanol can be an excellent alternative for the CSE of edible oil from *A. domesticus* using *n*-hexane.

## 4. Conclusions

The current work evaluated the combined potential of optimal ultrasonication and selected organic solvent to improve the extraction yield and quality of LMO. The initial assessment of LMOs extracted by pure and binary mixed organic solvents showed that the use of ethanol/isopropanol resulted in a good EE. Second-order polynomial models constructed in RSM-CCRD revealed sufficient reliability in predicting the EE and antioxidant activity of extracted LMOs during the UAE. The UAE time of 22.64 min, UAE temperature of 70 °C, and the solvent-to-LMLPs ratio of 22.5 *v*/*w* were selected and applied for effective extraction. Satisfactory EE, SC_DPPH_, and RP of LMO were achieved using the optimal conditions. The antioxidant potential of LMOs strongly depended on bioactive compounds such as carotenoids and phenolics. A comparison between CSE (with *n*-hexane) and UAE (with ethanol/isopropanol) methods revealed that the optimal ultrasonication process significantly produced LMO with a higher EE and bioactivity under the reduced EEC. Ultrasonication caused an extensive structural rupture of LMLPs and improved the quick transfer of oil from external and internal parts into ethanol/isopropanol as an effective substituent of *n*-hexane. Therefore, the diffusivity of oil in the selected solvent was remarkably increased by acoustic waves. The ultrasound extracted LMOs with more thermal stability without any significant effect on the main dominant fatty acids. More in-depth studies are in progress on the kinetic behavior of LMO-UAE. However, the optimized ultrasonic approach is satisfactory for obtaining high-quality oils from other insects with at least similar cell structures to lesser mealworms. Since consuming less solvent for the UAE makes it economically reasonable and feasible for industrial use, more effort should be directed to reducing the volume of solvent used in the extraction process. It is recommended that other bioactive minor compounds (e.g., tocopherols) be analyzed, in addition to the profile of carotenoids and phenolic compounds, and the content and type of polar lipids present in LMOs extracted by different solvents and techniques.

## Figures and Tables

**Table 1:** RSM-CCRD and experimental and predicted results for response variables.

Trial	Independent Variables			Response Variables		
	UAE Time (min, X_1_)	Solvent/Solid (*v*/*w*, X_2_)	UAE Temperature (°C, X_3_)	EE (%, Y_1_)	SC_DPPH__⋅_ (%, Y_2_)	RP (Y_3_)
1	15 (−1, Factorial)	7.5 (−1, Factorial)	30 (−1, Factorial)	37.6 ± 1.2	45.0 ± 0.6	0.326 ± 0.010
2	30 (+1, Factorial)	7.5 (−1, Factorial)	30 (−1, Factorial)	56.0 ± 2.4	58.1 ± 0.4	0.417 ± 0.014
3	5 (−1, Factorial)	22.5 (+1, Factorial)	30 (−1, Factorial)	68.9 ± 1.8	66.6 ± 0.3	0.532 ± 0.009
4	15 (−1), Factorial	22.5 (+1, Factorial)	30 (−1, Factorial)	79.5 ± 1.0	69.3 ± 1.0	0.666 ± 0.001
5	30 (+1, Factorial)	7.5 (−1, Factorial)	70 (+1, Factorial)	63.0 ± 1.0	56.6 ± 0.3	0.432 ± 0.004
6	5 (−1, Factorial)	7.5 (−1, Factorial)	70 (+1, Factorial)	78.1 ± 2.0	64.5 ± 1.1	0.509 ± 0.012
7	15 (−1, Factorial)	22.5 (+1, Factorial)	70 (+1, Factorial)	84.7 ± 2.2	69.3 ± 0.7	0.575 ± 0.007
8	30 (+1, Factorial)	22.5 (+1, Factorial)	70 (+1, Factorial)	92.3 ± 1.3	60.4 ± 0.5	0.532 ± 0.007
9	9.89 (−α, Axial)	15 (0, Center)	50 (0, Center)	65.5 ± 0.6	40.0 ± 0.8	0.274 ± 0.005
10	35.11 (+α, Axial)	15 (0, Center)	50 (0, Center)	85.4 ± 0.7	58.1 ± 1.7	0.434 ± 0.006
11	22.5 (0, Center)	2.39 (−α, Axial)	50 (0, Center)	51.6 ± 1.0	56.4 ± 1.5	0.402 ± 0.013
12	22.5 (0, Center)	27.61 (+α, Axial)	50 (0, Center)	90.4 ± 1.2	78.4 ± 1.1	0.796 ± 0.001
13	22.5 (0, Center)	15 (0, Center)	16.36 (−α, Axial)	41.0 ± 1.5	65.3 ± 0.8	0.632 ± 0.003
14	22.5 (0, Center)	15 (0, Center)	83.64 (+α, Axial)	80.2 ± 1.2	73.9 ± 0.8	0.686 ± 0.013
15	22.5 (0, Center)	15 (0, Center)	50 (0, Center)	73.3 ± 0.7	59.5 ± 1.2	0.479 ± 0.014
16	22.5 (0, Center)	15 (0, Center)	50 (0, Center)	76.9 ± 0.4	60.3 ± 0.6	0.488 ± 0.009
17	22.5 (0, Center)	15 (0, Center)	50 (0, Center)	71.5 ± 0.9	63.5 ± 0.4	0.545 ± 0.005
18	22.5 (0, Center)	15 (0, Center)	50 (0, Center)	75.4 ± 1.3	62.3 ± 0.5	0.492 ± 0.004
19	22.5 (0, Center)	15 (0, Center)	50 (0, Center)	73.4 ± 1.9	63.1 ± 0.3	0.535 ± 0.004
20	22.5 (0, Center)	15 (0, Center)	50 (0, Center)	69.9 ± 1.0	61.3 ± 0.8	0.529 ± 0.002

**Table 2:** ANOVA table for the experimental variables of each response variable and corresponding coefficients for the predictive models.

Source	DF	EE (%, Y_1_) ^1^			SC_DPPH__⋅_ (%, Y_2_) ^1^			RP (Y_3_) ^1^		
		C	SS	*p*-Value	C	SS	*p*-Value	C	SS	*p*-Value
**Model ^2^**	9	73.37	4152.90	<0.0001	61.72	1357.42	<0.0001	0.51	0.26	<0.0001
X_1_	1	6.28	539.28	<0.0001	3.31	149.35	0.0010	0.039	0.02	0.0076
X_2_	1	11.40	1773.81	<0.0001	5.74	449.44	<0.0001	0.094	0.12	<0.0001
X_3_	1	10.37	1469.62	<0.0001	1.93	50.78	0.0230	-	0.01	0.2409 ^ns^
X_1_^2^	1	-	14.29	0.1377 ^ns^	−4.63	308.65	<0.0001	−0.064	0.06	0.0002
X_2_^2^	1	-	4.63	0.3800 ^ns^	1.84	49.00	0.0249	-	0.007	0.0748 ^ns^
X_3_^2^	1	−4.25	260.41	<0.0001	2.63	99.72	0.0037	0.044	0.028	0.0031
X_1_X_2_	1	−1.97	31.17	0.0385	−3.39	91.89	0.0048	-	0.002	0.5403 ^ns^
X_1_X_3_	1	-	5.53	0.3393 ^ns^	−2.09	35.07	0.0499	-	0.0007	0.1469 ^ns^
X_2_X_3_	1	−2.31	42.74	0.0191	−3.02	73.14	0.0092	−0.036	0.004	0.0386
Residual	10		54.91			70.55			0.018	
LoF ^1^	5		22.88	0.6395 ^ns^		58.13	0.0578 ^ns^		0.014	0.0911 ^ns^
Pure error	5		32.03			12.42			0.004	
Total	19		4207.81			1427.97			0.28	
R^2^		0.9870			0.9506			0.9348		
R^2^_adj_		0.9752			0.9061			0.8760		
CV		3.31			4.31			8.35		
AP		33.86			18.36			15.41		

^1^ DF—Degree of Freedom, C—Coefficient, SS—Sum of squares, ns—non-significant, LoF—Lack-of-fit. ^2^ X_1_—UAE time, X_2_—solvent-to-LMLPs ratio, and X_3_—UAE temperature.

**Table 3:** The process efficiencies, bioactive compounds, antioxidant activities, fatty acids composition, and physicochemical properties of LMOs extracted by CSE and UAE methods.

Property	Extraction Method ^1,2^	
	CSE with *n*-Hexane	UAE with Ethanol/Isopropanol
**Process efficiency, energy, and diffusion coefficient**		
Extraction efficiency (%)	60.09 ± 1.32 ^b^	89.41 ± 1.87 ^a^
Diffusion coefficient (D, ×10^−9^ m^2^/s)	5.07 × 10^−11 b^	0.97 ± 0.05 × 10^−9 a^
Electric energy consumption (EEC, kW.h/g)	0.647 ± 0.009 ^a^	0.035 ± 0.003 ^b^
**Bioactive compounds and antioxidant activity**		
Total carotenoid content (TCC, mg/g)	0.645 ± 0.044 ^b^	0.778 ± 0.032 ^a^
Total phenolic content (TPC, mg GAE/g)	3.652 ± 0.015 ^b^	4.306 ± 0.029 ^a^
DPPH scavenging capacity (SC_DPPH__⋅,_ %)	60.10 ± 2.32 ^b^	71.31 ± 0.84 ^a^
Reducing power (RP)	0.517 ± 0.012 ^b^	0.663 ± 0.016 ^a^
**Physicochemical properties**		
Browning index (BI)	0.316 ± 0.009 ^a^	0.299 ± 0.05 ^a^
Photometric color index (PCI)	14.98 ± 0.09 ^a^	15.22 ± 0.11 ^a^
Specific gravity	0.9005 ± 0.0003 ^a^	0.9003 ± 0.0003 ^a^
Refractive index	1.452 ± 0.003 ^a^	1.450 ± 0.002 ^a^
Apparent visocisty (cP)	300.78 ± 5.62 ^a^	300.29 ± 7.01 ^a^
Acid value (AV, mg KOH/g)	1.78 ± 0.09 ^a^	1.67 ± 0.10 ^b^
Saponification value (SV, mg KOH/g)	221.05 ± 1.35 ^a^	220.35 ± 0.19 ^a^
Peroxide value (PV, meq O_2_/kg)	0.303 ± 0.009 ^a^	0.269 ± 0.011 ^b^
p-Anisidine value (AnV)	0.201 ± 0.04 ^a^	0.193 ± 0.02 ^b^
Totox value (TxV)	0.807 ± 0.005 ^a^	0.731 ± 0.005 ^b^
Iodine value (IV, g iodine/100 g)	88.70 ± 0.71 ^a^	83.52 ± 0.34 ^b^
Conjugated diene (K_232_)	1.64 ± 0.07 ^a^	1.55 ± 0.03 ^b^
Conjugated triene (K_270_)	0.126 ± 0.005 ^a^	0.109 ± 0.004 ^b^
Induction time (IT, h)	17.22 ± 0.61 ^b^	19.56 ± 0.40 ^a^
Free fatty acid (FFA, mg/kg)	0.34 ± 0.04 ^a^	0.24 ± 0.03 ^b^
**Fatty acids profile**		
Caproic acid (C6:0)	0.15 ± 0.02 ^a^	0.15 ± 0.01 ^a^
Lauric acid (C12:0)	0.32 ± 0.02 ^a^	0.29 ± 0.01 ^a^
Myristic acid (C14:0)	0.91 ± 0.03 ^a^	0.93 ± 0.07 ^a^
Palmitic acid (C16:0)	28.65 ± 0.26 ^a^	26.32 ± 0.18 ^a^
Palmitoleic acid (C16:1)	0.30 ± 0.02 ^a^	0.28 ± 0.00 ^a^
Stearic acid (C18:0)	7.68 ± 0.06 ^a^	7.56 ± 0.38 ^a^
Oleic acid (C18:1Δ9c)	28.01 ± 0.76 ^a^	29.54 ± 0.25 ^a^
Linoleic acid (C18:2n-6)	30.18 ± 0.44 ^a^	31.52 ± 0.63 ^a^
α-Linolenic acid (C18:3n-3)	2.05 ± 0.11 ^a^	2.01 ± 0.05 ^a^
Arachidic acid (C20:0)	0.31 ± 0.05 ^a^	0.30 ± 0.02 ^a^
Gondoic acid (C20:1Δ11)	0.38 ± 0.03 ^a^	0.34 ± 0.01 ^a^

^1^ CSE and UAE are convention solvent extraction (with *n*-hexane, 120 min time, 65 °C temperature, solvent/solid ratio of 10.0:1 *v*/*w*) and ultrasound-assisted extraction (with ethanol/isopropanol under the optimal conditions (22.64 min time, 70.0 °C temperature, solvent/solid ratio of 22.5:1 *v*/*w*); ^2^ Differences in treatment means in each row with the same statistical letter (a,b) are statistically non-significant.

## Data Availability

Not applicable.
